# Potential benefits of public–private partnerships to improve the efficiency of urban wastewater treatment

**DOI:** 10.1038/s41545-023-00232-2

**Published:** 2023-02-20

**Authors:** Shulei Cheng, Yu Yu, Fanxin Meng, Jiandong Chen, Yongtao Chen, Gengyuan Liu, Wei Fan

**Affiliations:** 1 School of Public Administration, Southwestern University of Finance and Economics, Chengdu, 611130 China; 2 State Key Joint Laboratory of Environment Simulation and Pollution Control, School of Environment, Beijing Normal University, Beijing, 100875 China; 3 School of Finance, Southwestern University of Finance and Economics, Chengdu, 611130 China; 4 School of Economics, Southwestern University of Finance and Economics, Chengdu, 611130 China

**Keywords:** Environmental sciences, Economics, Water resources

## Abstract

For emerging economies lacking public budgets, continuous improvement of urban wastewater treatment efficiency (UWTE) requires effective government supervision of wastewater treatment infrastructures (WTIs) and participation of private capital seeking to profit-maximising. However, to what extent this public–private partnership (PPP) model, aimed at a reasonable sharing of benefit and risk, in delivering WTIs can improve the UWTE is unknown. We evaluated the impact of the PPP model on the UWTE by collecting data from 1303 urban wastewater treatment PPP projects in 283 prefecture-level cities in China from 2014 to 2019 and used data envelopment analysis and Tobit regression model. The UWTE was significantly higher in prefecture-level cities that introduced the PPP model in the construction and operation of WTIs, particularly those with a feasibility gap subsidy, competitive procurement, privatised operation, and non-demonstration. Moreover, the effects of PPPs on UWTE were limited by the economic development level, marketisation, and climatic conditions.

## Introduction

Urban wastewater treatment capacity in emerging economies is insufficient for sustainable production and living^1^. The industrial agglomeration and population growth in urban areas inevitably generate increasing volumes of wastewater. However, the limited public budgets of governments force demands improving the urban wastewater treatment efficiency (UWTE) rather than expanding wastewater treatment infrastructures (WTIs) globally^2^, particularly following multiple of global economic crises and the ongoing coronavirus pandemic (COVID-19). In a city, UWTE usually refers to the amount of urban wastewater that can be purified by WTIs at a given scale^3^. Improving the UWTE entails treating as much wastewater as possible to meet the required standards at the lowest cost regarding personnel, capital, and equipment at a given level of technology^4^. Improvements in the UWTE are important aspects of enhancing urban environmental quality^5^, protecting human health^6^, and achieving multiple Sustainable Development Goals in a country^7^.

Governments worldwide use various tools, including penalties, subsidies, and administrative rules, to inhibit the discharge-based wastewater^8^. Nevertheless, strengthening the discharge-based supervision does not mean the inevitable improvement of the UWTE. Once the urban wastewater has been produced, the ability of the WTI to purify it becomes the key element in determining the UWTE. Actually, governments have difficulty forming an inspection deterrent for WTIs and cannot effectively regulate them^9^. The complexity of bureaucracy, including the policy design, implementation, and supervision functions of urban wastewater treatment, and the boundaries between the roles of government departments are blurred, resulting in a limited improvement of the UWTE by the government’s unilateral regulation^10^. Therefore, enhancing the WTI capacity to enable it to purify urban wastewater, thereby improving the UWTE is critical.

Efforts have been made to link macro-institutions and policies to the WTI capacity, for example, the river chief system^11^, diversified financing mechanisms and professional management systems^12^, community associations institution^13^, preventive maintenance policy^14^, and sharing and eco-industrial park policies^15,16^. However, the construction and operation mode selected for the WTI behind the macro-institutions and policies is lack of understanding.

Those countries that advocate a service-oriented government tend to entrust state-owned enterprises to build and operate WTIs to ensure the right of enterprises and households to use clean water. This can guarantee clean water at a lower price with the support of fiscal subsidies; however, it is difficult for cities with few fiscal budgets^17^. In contrast, private-owned WTIs that seek to profit-maximising can improve the economic efficiency and performance;^18^ however, the economic efficiency and performance are frequently distorted (accurately determining the high monopolistic cost of clean water^19^), and the public value is hard to fully embody. Moreover, the government has difficulty in effectively supervising private urban wastewater treatment enterprises; for example, water quality, health standards, and wastewater treatment plans constantly change, and thus, monitoring multiple aspects of wastewater treatment (e.g. bulk water collection and storage, wastewater collection, distribution of water, and water treatment) is practically complicated^20^.

The PPP model, which advocates an equal distribution of risk and benefit, is recently emerging as a new model to replace public and private WTIs^21^. Through the collaboration of government and private enterprises, contracts for the construction and operation of urban wastewater treatment facilities are signed, meaning both parties can share risks and profits. Previous scholars have adopted the descriptive case analysis^22^, performance assessment rating tool^23^, process management life cycle performance measurement system^24^, analytical hierarchy process^25^, observational study^26^, and fuzzy set qualitative comparative analysis^27^ to explore how the public service efficiency under the PPP model can be quantified.

With many emerging economies vigorously promoting PPP model in urban infrastructures such as transportation, energy services, water and sewage, public health, and environment protection^28^, there is increasing pressure to verify whether urban infrastructure introduced with PPP mode would also have a high efficiency. For wastewater, Beisheim and Campe^29^ and Tang et al.^10^ have sequentially confirmed that a high level of institutionalisation (obligation, precision, and delegation) and technological advantages are key to significantly improving the service efficiency of urban water utilities. Although they have confirmed the high wastewater treatment capacity of a single urban wastewater treatment enterprise under the PPP model, the potential impact of the PPP model on the overall UWTE at the city level has been unexplored. Thus, a systematic analysis of whether a single urban wastewater treatment enterprise adopting the PPP model can improve the citywide UWTE is needed.

To address these knowledge gaps, the impacts of PPP models on the UWTE were assessed using the Tobit regression model. We collected data on 1303 urban wastewater treatment PPP projects in 283 cities of the Chinese prefecture from 2014 to 2019 and matched them in the prefecture-level city dimension with the UWTE data derived from the data envelopment analysis (DEA). This study was aimed to resolve the following research questions: (1) Is the UWTE significantly higher in prefecture-level cities, that have implemented PPPs to build and operate WTIs, than in those that have not? (2) For prefecture-level cities that have introduced PPPs, which return, procurement, and operation mechanisms are optimal for urban wastewater treatment? (3) Does access to the PPP demonstration project help improve the UWTE? (4) The study provides further insights and policy implications on the construction and operation of WTIs in cities in emerging economies that lack public budgets.

## Results

### PPP models for urban wastewater treatment and the UWTE in China

**Fig. 1:**
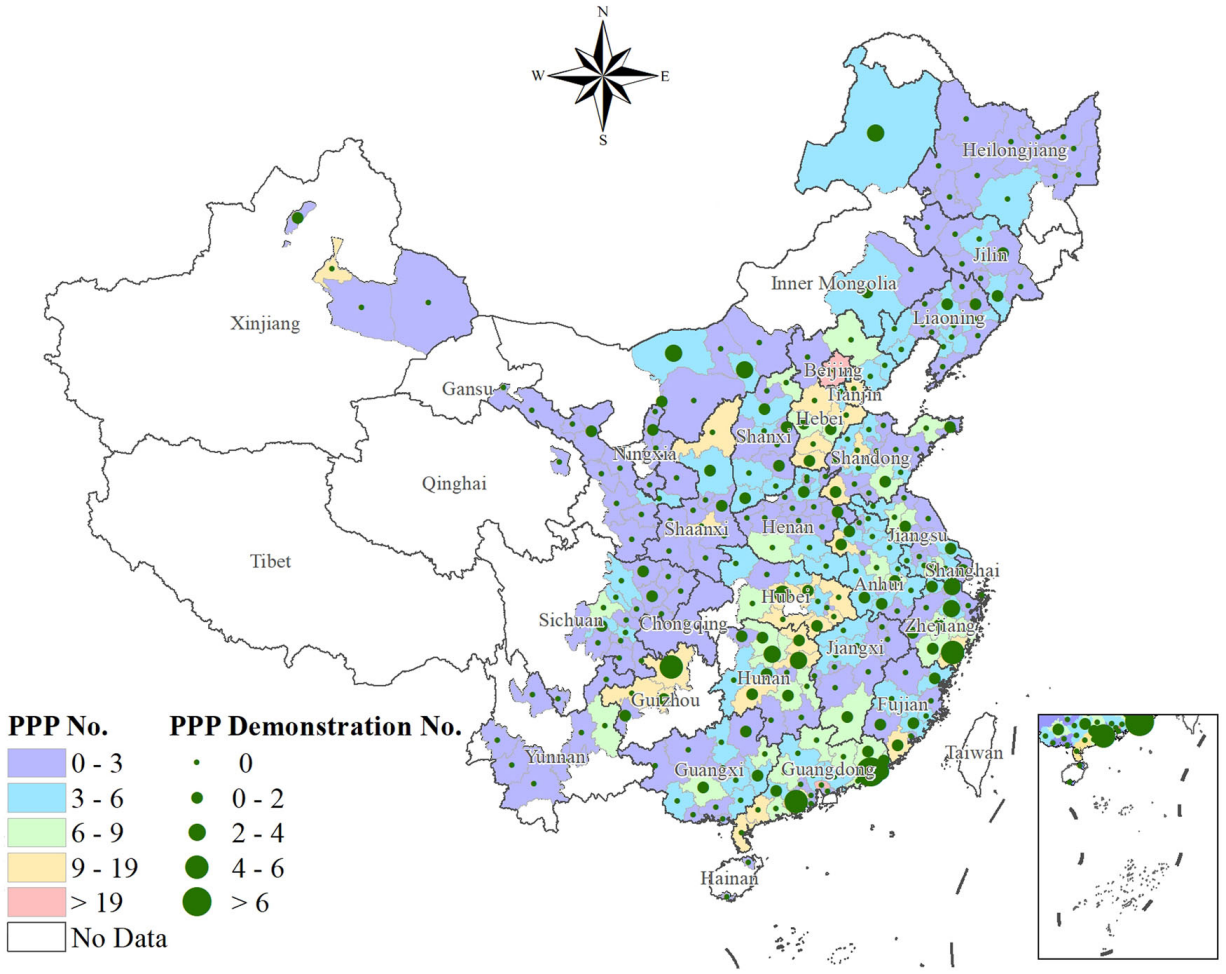
PPPs for wastewater treatment in Chinese prefecture-level cities. The data of the PPP projects at the end of 2019 is shown. PPP No. represents the number of PPP projects for wastewater treatment, and the colour from purple to red represents the number of projects from small to large. The critical value takes the colour which belongs to the higher interval. PPP Demonstration No. represents the number of PPP demonstration projects for wastewater treatment, and the green dots from small to large represent the number of demonstration projects from small to large. The critical value takes the dot scale which belongs to the higher interval. The division of the research area is based on prefecture-level cities. The uncoloured prefecture-level cities lack data on PPP projects.

In 2014, the central government of China suggested local governments to encourage private investors to participate in the construction and operation of urban infrastructure. As a critical urban infrastructure, wastewater treatment is the first to introduce PPPs, and it has the largest number of PPP projects; the wastewater treatment projects represented 43% of all the PPP projects by the end of 2019. Furthermore, 253 prefecture-level cities have introduced PPPs in WTIs, representing 89% of all prefecture-level cities. The wastewater treatment PPP projects of 75 of these prefecture-level cities are included in the demonstration projects (Fig. 1). In terms of return mechanisms, user payment, feasibility gap subsidy, and government payment accounted for 14%, 44%, and 42%, respectively. In terms of procurement mechanisms, competitive and non-competitive states accounted for 95% and 5%, respectively. In terms of operation mechanisms, outsourcing, franchising, and privatised operation accounted for 0.8%, 0.3%, and 82.6%, respectively.

**Fig. 2:**
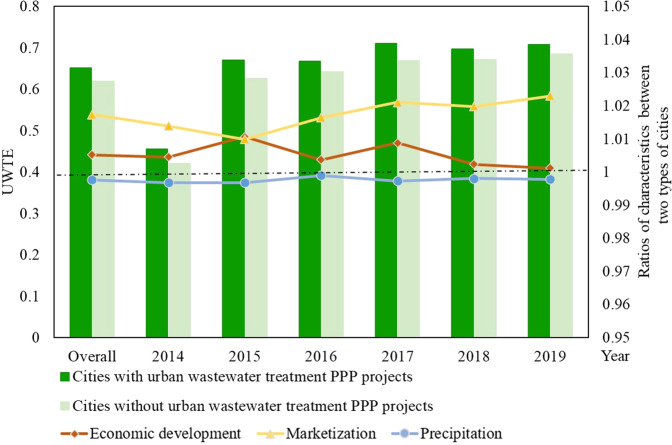
Average UWTE with/without PPPs for wastewater treatment and comparison of characteristics in Chinese prefecture-level cities. The average UWTE from 2014–2019 in Chinese prefecture-level cities is shown. The height of the column represents the UWTE, and the higher the column, the larger the UWTE. The scattered dots of different colours represent the ratio of cities’ characteristics with urban wastewater treatment PPP projects to cities that without urban wastewater treatment PPP projects. The scattered dots of red, yellow and blue represent the cities’ economic development level, marketization level and precipitation, respectively. The horizontal black dashed line in the figure indicates that the ratio is 1, implying the same average conditions.

In general, prefecture-level cities that have introduced the PPP model for their WTIs had a higher UWTE (average of 0.651) than those that have not (average of 0.619) (Fig. 2). This feature also appears in the comparison results from year to year. In terms of prefecture-level city characteristics, the average economic development level and average marketisation of the prefecture-level cities that introduced the PPP model for WTIs were high, whereas the average precipitation of the prefecture-level cities that introduced the PPP model for WTIs were low.

### Impacts of PPPs on the UWTE at prefecture-level city scales

Table 1 reports the impact of PPPs on the UWTE. The explanatory variables in columns (1) and (2) represent the presence or absence of wastewater treatment PPP projects. The benchmark regression results show that when only the influence of the variable, ‘presence or absence of wastewater treatment PPP projects’, is considered, its regression coefficient is positively significant at the 1% level (*p* < 0.01) (first column of Table 1). Considering the influence of control variables such as population density, urbanisation, GDP per capita, industrialisation, openness, and green innovations, the coefficient of the variable, ‘presence or absence of wastewater treatment PPP projects’, was still positively significant at the 1% level (*p* < 0.01). The average UWTE of prefecture-level cities with the PPP model was 0.046 units higher than that of prefecture-level cities without the PPP model (second column of Table 1).

**Table 1:** Estimation results of the benchmark regression model.

Dependent variable	(1)	(2)	(3)	(4)
	Tobit	Tobit	Tobit	Tobit
PPP	0.067***	0.046***		
	(0.008)	(0.007)		
PPPn			0.014***	0.011***
			(0.002)	(0.002)
popdensity		−0.000		−0.000
		(0.000)		(0.000)
urban		0.000		0.000
		(0.001)		(0.001)
lngdp		0.088***		0.092***
		(0.013)		(0.013)
industrial		−0.005***		−0.005***
		(0.001)		(0.001)
openness		−0.001***		−0.001***
		(0.000)		(0.000)
lngreen		0.021***		0.022***
		(0.004)		(0.004)
Constant	0.603***	−0.166	0.618***	−0.184
	(0.007)	(0.124)	(0.007)	(0.125)
Obs.	1698	1668	1698	1668
Wald test	78.370	334.330	38.860	320.050
	(0.000)	(0.000)	(0.000)	(0.000)
Log Likelihood	725.603	833.148	706.574	828.308
Rho	0.337	0.466	0.332	0.469

The dependent variable is referred to by the value of UWTE. Marginal effects are reported in the table. Standard errors are in parentheses. ^***^ denotes significant levels of 1%. The *P*-values are reported in parentheses in the Wald test.

Owing to the number of wastewater treatment PPP projects partly reflecting the sustainability of the PPP model, explanatory variables in columns (3) and (4) represent the number of wastewater treatment PPP projects. The results show that the regression coefficients of the explanatory variables were robustly and positively significant at the 1% level (*p* < 0.01). As the control variables remain unchanged, each additional unit of the wastewater treatment PPP project, on average, increased the UWTE by 0.011 units (fourth column of Table 1).

Some control variables also gave valuable information. The regression coefficients of GDP per capita and green innovations were both robustly positive and significant at the 1% level, whereas those of the industrialisation and openness were both robustly negative and significant at the 1% level, which are both consistent with our expectations.

### Endogeneity check

To verify that the relationship between the PPPs and UWTE is causal rather than correlation, Table 2 presents the endogeneity tests between the PPPs and UWTE based on two-stage instrumental variable regressions. The instrumental variables selected in columns (1) and (2) represent the presence or absence of a waste treatment PPP project, those selected in columns (3) and (4) are the number of waste treatment PPP projects, and those in columns (5) and (6) represent the mean number of wastewater treatment PPP projects in neighbouring prefecture-level cities in the province where the prefecture-level city is located. Columns (1), (3), and (5) resulted from the second-stage of the instrumental variable regression, and columns (2), (4), and (6) resulted from the first-stage of the instrumental variable regression. The instrumental variable regression results usually should pass a series of tests, and all the instrumental variables selected in this study passed the weak instrumental variable test^30^. The regression coefficients that determine the presence or absence of a wastewater treatment PPP project and number of wastewater treatment PPP projects in the second-stage regressions and the presence or absence of a waste treatment PPP project, number of waste treatment PPP projects, and mean number of wastewater PPP projects in neighbouring prefecture-level cities in the province in the first-stage regressions were all robustly and positively significant at the 1% level. These checks removed the potential endogeneity problem between the PPPs and UWTE. This implies that the adoption of PPPs in the construction and operation of WTIs affects the UWTE and can effectively promote the transformation of urban wastewater treatment.

**Table 2:** Robustness check results.

Dependent variable	(1)	(2)	(3)	(4)	(5)	(6)	(7)	(8)
			Second Stage	First Stage	Second Stage	First Stage	Second Stage	First Stage
lnPPPinv	0.006***	0.004***			0.017***		0.013***	
	(0.001)	(0.001)			(0.002)		(0.004)	
PPPn			0.054***					
			(0.006)					
meanPPPn				0.999***				
				(0.050)				
wastePPPinv						0.984***		
						(0.041)		
meanPPPinv								0.280***
								(0.031)
Controls	NO	YES	YES	YES	YES	YES	YES	YES
Constant	0.611***	−0.171	0.191^*^	1.643^*^	0.233**	2.684	0.226**	−0.741
	(0.007)	(0.125)	(0.112)	(0.981)	(0.110)	(2.581)	(0.107)	(2.926)
Obs.	1698	1668	1668		1668		1668	
Wald test	55.020	320.130	193.090		200.740		128.610	
	(0.000)	(0.000)	(0.000)		(0.000)		(0.000)	
Log Likelihood	714.399	828.172	−2429.263		−4045.351		−4294.964	
Rho	0.337	0.471						
First stage F statistics				63.580		98.050		22.690
				(0.000)		(0.000)		(0.000)

In columns (1) and (2), the dependent variable is referred to by the value of UWTE. In the first stage, the dependent variable is referred to by the value of UWTE, and in the second stage, the dependent variable is the corresponding variable that may not be robust. Marginal effects are reported in the table. Standard errors are in parentheses. ^***, **, *^ denote significant levels of 1%, 5%, and 10%, respectively. *P*-values are reported in parentheses in the Wald test and First stage F statistics.

### Robustness check

Table 3 presents the robustness check results. Column (1) replaces the presence or absence of a wastewater treatment PPP project and the number of wastewater treatment PPP projects in the benchmark regression with the investment amount of wastewater treatment PPP projects. Although both are commonly used indicators to measure the extent of PPP development, the investment amount of PPP projects is sometimes used because the potential impact of PPPs on the scale and quality of urban infrastructure supply is ultimately reflected in the investment amount^31^. Column (2) determines the effects of control variables such as population density, urbanisation, GDP per capita, industrialisation, openness, and green innovations based in column (1). Columns (3) and (4) replace the mean number of wastewater treatment PPP projects in neighbouring prefecture-level cities in the province (excluding the prefecture-level city) with the mean number of wastewater treatment PPP projects in the province (including the prefecture-level city) in the original instrumental variable regression. Furthermore, columns (5) and (6) replace the mean number of wastewater treatment PPP projects in neighbouring prefecture-level cities in the province (excluding the prefecture-level city) in the original instrumental variable regression with the mean investment amount of wastewater treatment PPP project in the province (including the prefecture-level city); columns (3) and (5) resulted from the second-stage of the instrumental variable regression, and columns (4) and (6) resulted from the first-stage of the instrumental variable regression. This eliminated the complexity of obtaining information on the PPP development of other neighbouring prefecture-level cities for certain prefecture-level cities when considering the construction and operation of WTI and the difficulty of relying only on the mean level of PPP development of their province. Furthermore, columns (7) and (8) used the investment amount of the waste treatment PPP projects to determine the presence or absence and number of projects in the original instrumental variable regression, where the result of the former was the second-stage of the instrumental variable regression, and the latter was the first-stage result of the instrumental variable regression. After several checks, the potential impact of PPPs on the UWTE remained robust and positively significant (*p* < 0.01).

**Table 3:** Estimation results of the instrument variable regression model using 2SLS estimations method.

Dependent variable	(1)	(2)	(3)	(4)	(5)	(6)
	Second Stage	First Stage	Second Stage	First Stage	Second Stage	First Stage
PPP	0.227***					
	(0.040)					
PPPn			0.038***		0.116***	
			(0.014)		(0.014)	
wastePPP		0.256^***^				
		(0.032)				
wastePPPn				0.583***		
				(0.080)		
meanPPPn						0.595***
						(0.059)
Controls	YES	YES	YES	YES	YES	YES
Constant	0.187	0.166	0.194^*^	0.491	0.185	1.182
	(0.116)	(0.298)	(0.107)	(1.072)	(0.152)	(1.052)
Obs.	1668	1668	1668	1668	1638	1638
Wald test	132.230		125.480		123.450	
	(0.000)		(0.000)		(0.000)	
Log Likelihood	−423.402		−2618.411		−2473.686	
First stage F statistics		18.820		13.670		20.550
		(0.000)		(0.000)		(0.000)

In the first stage, the dependent variable is referred to by the value of UWTE, and in the second stage, the dependent variable is the corresponding variable that may be endogenous. Marginal effects are reported in the table. Standard errors are in parentheses. ^***, *^ denote significant levels of 1% and 10%, respectively. *P* values are reported in parentheses in the Wald test and first stage F statistics.

### Roles of institutional mechanisms in the effects of PPPs on UWTE

Tables 4–7 introduce the institutional mechanisms of the PPP model affect the UWTE in terms of return, procurement, operation, and demonstration. Table 4 presents the results of the return mechanism in the PPP model that affect the UWTE. Columns (1) to (3) indicate the regression results under the feasibility gap subsidy, user payment, and government payment mechanisms. The regression results show that after adding the influence of control variables, although the PPP projects in all return mechanisms significantly and positively affected the UWTE (*p* < 0.01, *p* < 0.05, *p* < 0.01, respectively), the effects of the PPP projects with the feasibility gap subsidy mechanism on the UWTE exceeded that under the other two mechanisms. The regression coefficients indicate that for each additional unit of the wastewater treatment PPP project with the feasibility gap subsidy return mechanism, on average, the UWTE increased by 0.023 units. However, each additional unit of the wastewater treatment PPP project with the government payment and user payment return mechanisms increased the UWTE by only 0.01 and 0.007 units, respectively, on average.

**Table 4:** Effect of the return mechanism.

Dependent variable	(1)	(2)	(3)
	Feasibility gap subsidy	Government payment	User payment
PPPn	0.023***	0.010***	0.007**
	(0.004)	(0.004)	(0.003)
Controls	YES	YES	YES
Constant	−0.180	−0.202	−0.239*
	(0.125)	(0.126)	(0.126)
Obs.	1668	1668	1668
Wald test	320.400	290.600	286.190
	(0.000)	(0.000)	(0.000)
Log Likelihood	827.870	817.071	815.744
Rho	0.466	0.465	0.473

The dependent variable is referred to by the value of UWTE. Marginal effects are reported in the table. Standard errors are in parentheses. ^***, **, *^ denote significant levels of 1%, 5%, and 10%, respectively. The *P* values are reported in parentheses in the Wald test.

**Table 5:** Effect of the procurement mechanism.

Dependent variable	(1)	(2)
	Single-source procurement	Competitive procurement
PPPn	0.011	0.011***
	(0.022)	(0.002)
Controls	YES	YES
Constant	−0.231*	−0.184
	(0.126)	(0.125)
Obs.	1668	1668
Wald test	280.900	318.310
	(0.000)	(0.000)
Log Likelihood	813.513	827.555
Rho	0.470	0.468

The dependent variable is referred to by the value of UWTE. Marginal effects are reported in the table. Standard errors are in parentheses. ^***, *^ denote significant levels of 1% and 10%, respectively. The *P* values are reported in parentheses in the Wald test.

**Table 6:** Effect of the operation mechanism.

Dependent variable	(1)	(2)	(3)
	Outsourcing	Franchising	Privatised operation
PPPn	0.040	−0.031	0.011***
	(0.038)	(0.067)	(0.002)
Controls	YES	YES	YES
Constant	−0.232*	−0.230*	−0.193
	(0.126)	(0.126)	(0.125)
Obs.	1668	1668	1668
Wald test	281.840	280.870	309.850
	(0.000)	(0.000)	(0.000)
Log Likelihood	813.931	813.485	824.340
Rho	0.470	0.469	0.468

The dependent variable is referred to by the value of UWTE. Marginal effects are reported in the table. Standard errors are in parentheses. ^***, *^ denote significant levels of 1% and 10%, respectively. The *P* values are reported in parentheses in the Wald test.

**Table 7:** Effect of demonstration.

Dependent variable	(1)	(2)
	Non-demonstration	Demonstration
PPPn	0.011***	0.008*
	(0.002)	(0.004)
Controls	YES	YES
Constant	−0.171	−0.241*
	(0.125)	(0.126)
Obs.	1668	1668
Wald test	313.790	284.770
	(0.000)	(0.000)
Log Likelihood	825.454	815.263
Rho	0.465	0.472

Notes: The dependent variable is referred to by the value of UWTE. Marginal effects are reported in the table. Standard errors are in parentheses. ^***,^^*^ denote 1% and 10%, respectively. The *P* values are reported in parentheses in the Wald test.

Table 5 presents the results of the procurement mechanism in the PPP model that affect the UWTE. Columns (1) and (2) indicate the regression results under single-source procurement and competitive procurement, respectively. The regression results show that after adding the influence of control variables, only the PPP projects with competitive procurement significantly and positively affected the UWTE (*p* < 0.01); whereas, the PPP projects under single-source procurement could not significantly improve the UWTE. The regression coefficients indicate that for each additional unit of the wastewater treatment the PPP project procured competitively, the UWTE increased by 0.011 units, on average.

Table 6 presents the results of the operation mechanism in the PPP model that affect the UWTE. Columns (1) to (3) indicate the regression results for the outsourcing, franchising, and privatisation modes of operation, respectively. The regression results show that after adding the influence of the control variables, only the PPP projects in the privatisation mechanism of operation significantly and positively affected UWTE (*p* < 0.01), and the PPP projects under the outsourcing or franchising mechanism significantly affected the UWTE. The regression coefficients indicate that for each additional unit of wastewater treatment, the PPP project operated under privatisation increased the UWTE by 0.011 units, on average.

Table 7 presents the results of demonstration or non-demonstration in the effect of the PPP model on the UWTE. Columns (1) and (2) indicate the regression results for the demonstration and non-demonstration categories, respectively. The regression results show that after adding the influence of the control variables, regardless of whether it is a demonstration project, the PPPs had a significant positive impact on the UWTE (*p* < 0.1, *p* < 0.01). Moreover, the effect of the PPPs on the UWTE that has not been demonstrated exceeded that of those that have been demonstrated. The regression coefficients show that for each additional unit of the demonstrated wastewater treatment PPP project, the UWTE increased by 0.008 units on average; however, for each additional unit of the wastewater treatment PPP project not demonstrated, the UWTE increased by 0.011 units on average.

### Heterogeneity of economic development, marketisation, and climatic conditions

Table 8 presents the results of the heterogeneous effects of PPPs on the UWTE in terms of economic development level, degree of marketisation, and climatic conditions. Columns (1), (2), and (3) indicate the regression results for the eastern, central, and western regions, respectively. There is a significant gap in the levels of economic development of the three regions, with the central region lagging the eastern region and the western region lagging the former. To promote the equalisation of urban public services among regions, the central government will prioritise regions with relatively backward economic development levels for the infrastructure PPP project approval policies^32^. These regions, which are otherwise disadvantaged in terms of capital, management experience, and technology, can therefore use the PPPs to improve the quality of public services, including the UWTE. The regression results show that although the PPP projects in the three regions significantly and positively affected UWTE (*p* < 0.01, *p* < 0.01, *p* < 0.05), the effect of the PPP projects on the UWTE in the western region exceeded that in the central region, which exceeded that of the eastern region. The regression coefficients show that for each additional unit of the wastewater treatment PPP project in the western region, the UWTE increased by 0.024 units on average; however, for each additional unit of in the central and eastern regions, the UWTE increased only by 0.021 and 0.008 units, respectively.

**Table 8:** Heterogeneous analysis.

Dependent variable	(1)	(2)	(3)	(4)	(5)	(6)	(7)
	East	Central	West	High level of marketisation	Low level of marketisation	Less precipitation	More precipitation
PPPn	0.008***	0.021***	0.024**	0.009***	0.015	0.009***	0.016**
	(0.002)	(0.006)	(0.010)	(0.002)	(0.010)	(0.003)	(0.008)
Controls	YES	YES	YES	YES	YES	YES	YES
Constant	−0.771^***^	−0.729^***^	0.159	−0.735**	−0.470*	−0.506*	−0.116
	(0.277)	(0.230)	(0.193)	(0.319)	(0.277)	(0.264)	(0.310)
Obs.	640	543	485	515	490	498	498
Wald test	129.910	131.170	86.180	123.730	83.070	146.540	95.930
	(0.000)	(0.000)	(0.000)	(0.000)	(0.000)	(0.000)	(0.000)
Log Likelihood	372.31	286.500	192.372	305.626	217.167	286.616	221.570
Rho	0.581	0.410	0.428	0.612	0.558	0.491	0.558

The dependent variable is referred to by the value of UWTE. Marginal effects are reported in the table. Standard errors are in parentheses. ^***, **,^ and ^*^ denote 1%, 5%, and 10%, respectively. The *P* values are reported in parentheses in the Wald test. High and low levels of marketisation refer to the upper 30% and lower 30% of samples, respectively. More and less precipitation refer to the upper 30% and bottom 30% of samples, respectively.

Columns (4) and (5) indicate the regression results for regions with high and low marketisation, respectively. The market environment is a prerequisite for the willingness of private capital to participate in the provision of urban public services. Regions with a relatively high degree of marketisation typically have a relatively low rent-seeking phenomenon, and the government honours the benefits given to private capital in accordance with the PPP contract. This reduces the transaction costs of private capital participation in urban infrastructure construction and operation^33^, creating conditions for the full utilisation of PPPs to improve the quality of urban public services, including the UWTE. The regression results show that the PPPs in regions with high marketisation significantly improved the UWTE (*p* < 0.01), whereas the effect of PPPs on the UWTE in regions with low marketisation was not significant (*p* > 0.1). The regression coefficients indicate that for each additional unit of the wastewater treatment PPP project in regions with high marketisation, the UWTE increased by 0.009 units, on average.

Columns (6) and (7) indicate the regression results for regions with more and less precipitation, respectively. Wastewater treatment is influenced by both socioeconomic factors and climatic conditions. A portion of treated urban wastewater comes from precipitation. Therefore, climatic conditions such as precipitation can indirectly affect the UWTE. If other controls are kept constant, regions with less precipitation have relatively less urban wastewater to treat, and their urban wastewater treatment facilities experience less pressure regarding urban wastewater treatment. Regression results show that both regions with more and less precipitation significantly and positively affected UWTE (*p* < 0.01, *p* < 0.05); however, the effect of PPPs on the UWTE in regions with less precipitation exceeded that in regions with more precipitation. The regression coefficients show that for each additional unit of the wastewater treatment PPP project in regions with less precipitation, the UWTE increased by 0.016 units on average; however, in regions with more precipitation, the UWTE only increased by 0.009 units on average.

## Discussion

Our study revealed that the construction and operation of WTIs using the PPP model have improved the UWTE, and the more sustainable the application of PPP model in the urban wastewater treatment services, the greater the UWTE improvement. The conclusions are not only verified with the study on the PPPs to improve the energy infrastructure operation efficiency based on provincial data and stochastic frontier analysis^34^ but also corresponded with the study on the high service efficiency of urban water supply companies under the PPP model founded by enterprise data^10^. This suggests that the research has fully verified the incentive effect of the PPP model on public service efficiency, which is not only reflected in the enterprises that adopt the PPP model but also has a potential promotion effect on the citywide public service efficiency.

The results may aid local governments in China facing budget constraints to improve the UWTE. China is undergoing rapid urbanisation and industrialisation, accompanied by a surge in demand for urban wastewater treatment. Wastewater treatment facilities require vast public budgets during the construction and operation stages^35^, and the technology and consumer expectations for service quality are high^13^. Therefore, local government officials ‘take advantage of loopholes in the law’ to finance local infrastructure construction, for example, with land finance, local bonds, and local investment and financing platforms. However, the disordered development of these financing models has exposed local governments to significant potential debt risks when encouraging the construction of local infrastructure. Therefore, the promotion of the PPP model for urban wastewater treatment can be considered a way to improve the UWTE and avoid the excessive risk of implicit debt. Moreover, because our study specifically emphasises the identification of the causal relationship between the PPP model and UWTE, the empirical study of Chinese prefecture-level cities provides valuable insights for improving the UWTE in other emerging economies that are experiencing the contradiction between the need for urban expansion of public wastewater treatment services and limited local public budgets.

Notably, the results highlight the important role of four institutional mechanisms, return, procurement, operation, and demonstration, in the impacts of PPP models on the UWTE. Firstly, the PPP model with a feasibility gap subsidy return implies that appropriately sharing cost risk between government and private capital in the construction and operation of WTI is optimal for improving the UWTE. This further confirms the previous assertion that appropriate risk allocation is indispensable for improving large infrastructure construction and operation efficiency under PPPs^36^. Secondly, the PPPs with competitive procurement implies that only introducing a competitive mechanism in the procurement process can encourage innovation, cost savings, and efficiency. Private capital introduced in the single-source procurement process does not have the intrinsic motivation to pursue more efficient construction and operation of WTIs. This confirms the reason that previous studies have encouraged simplification and enhanced competitiveness in the PPP procurement process^37^. Thirdly, privatisation operation implies that giving private capitalists full autonomy in operating WTI is optimal for improving the UWTE. Recognising privatised operations such as Build-Own-Operate (BOO) and Buy-Build-Operate (BBO) could maximise the benefits promised in the PPP contract^38^, which is an incentive for private investors to improve the UWTE. Finally, the demonstrated PPP model is significant in drawing private capital parties to participate in the construction and operation of WTIs and improve the UWTE to the best of their ability. The selection of the wastewater treatment PPP project for demonstration implies priority support from government policies and funding and a relatively high possibility that private investors can obtain operational benefits from participation^39^. This is the reason the PPP projects that have not been demonstrated have a greater incentive effect on the UWTE than the PPP projects demonstrated.

Our study also shows that the level of economic development, degree of marketisation, and climatic conditions are the constraints that lead to the differentiated effects of the PPP model on the UWTE between prefecture-level cities. The PPPs can play a positive role in improving the UWTE in regions with a relatively backward economic development level with insufficient WTI, in regions with fully developed market with strong capacity to fulfil the contract, and in regions with more precipitation.

A few policy implications can be proposed: firstly, the government should further design and improve policies to guide social capital to participate in the supply of public services for urban wastewater treatment. We can increase the support for private capital to participate in these PPP projects by establishing and increasing the transfer payment funds related to wastewater treatment services from the central government. Stopping local governments from discriminating against the provision of public services for urban wastewater treatment without reason is also necessary. Secondly, encouraging the use of PPP models with a feasibility gap subsidy return mechanism, competitive procurement and privatisation, and leveraging the incentive role of demonstration PPP projects to attract social capital can improve the UWTE. Local governments can inject appropriate capital and operation subsidies to attract social capital to join in the provision of public services for urban wastewater treatment while improving the efficiency and ensuring the competitive procurement process and privatisation of operation methods from both institutional and legal aspects. Meanwhile, it can strengthen the management of demonstration PPP projects and provide superior profit-sharing contracts for non-demonstration wastewater treatment PPP projects to improve the UWTE of both types of projects. Thirdly, conditions can be created for the priority development of urban infrastructure projects under the PPP model in developing regions, for example regions with a low degree of marketisation and regions with less precipitation. For example, the Ministry of Finance prioritises supporting the storage of wastewater treatment PPP projects in these regions and encourages other regions to allocate horizontal special transfer funds to secure the necessary budgets for developing PPPs.

This study has some limitations. Firstly, we only used the data of wastewater treatment PPP projects up to 2019. This is because after 2019, China’s Ministry of Finance started to regulate PPPs development to solve the problems of local governments exceeding their own public budgets and solidifying government expenditure responsibilities. However, this treatment makes it difficult to judge the extent to which PPPs can affect the UWTE once the government’s attitude toward the PPP model changes. Secondly, we found that as long as a prefecture-level city adopts the PPP model to build and operate WTI, the citywide UWTE will be significantly improved; however, owing to the lack of survey data based on urban wastewater treatment enterprise level, how these enterprises that adopted the PPP model will drive the improvement of wastewater treatment efficiency in other enterprises that have not adopted this model is unknown. Finally, to verify whether the conclusions based on China are equally robust in other emerging economies, we should further collect more representative data from different countries and regions in the future and conduct research on the causal relationship between the PPPs and UWTE globally.

## Methods

### Study design and hypotheses

The encouragement of the Chinese central government to local governments to adopt the PPP model through the top-down procedure and build and operate WTI has created favourable external policy circumstances for the development of wastewater treatment PPP projects. However, the acceptance of the PPP model by both local governments and private capital is rooted in the positive effect of it on improving the UWTE. In China there is no completely private-owned WTI before. Compared to the original government monopoly on the construction and operation of the WTI, the introduction of private capital participation is equipped with conditions to improve the UWTE. The participation of private capital can donate sufficient funds, scientific management experience, and advanced technology to the construction and operation of the regional, quasi-natural monopoly, and public welfare WTI^21^, which are key elements that determine the UWTE. Furthermore, the urban wastewater treatment field was in a state of no market competition before the introduction of private capital, and the government’s early monopoly ensured that private capital could obtain both economic benefits and performance with exclusive agency rights after joining. Meanwhile, the government would conduct a performance assessment of the quality of wastewater treatment during construction and operation, and private capitals whose wastewater treatment efficiency failed to meet the requirements would be barred from obtaining performance benefits^40^. Therefore, private capital is inherently incentivised to ensure the UWTE and minimise profit loss. Most of the private capital involved in the construction and operation of WTI in China comes from state-owned enterprises, partly due to the remarkable cooperation between the local government and state-owned enterprises at the beginning of the market economy reform^41^. This is convenient for both sides in reducing the cost of supervising due to information asymmetry in the principal-agent relationship and to facilitate the unique advantages of state-owned capital to undertake social responsibility. Therefore, Hypothesis 1 is proposed:

UWTE is high in prefecture-level cities that have introduced the PPP model compared to prefecture-level cities that have not adopted the PPP model for the construction and operation of WTI.

The return mechanism is related to the risk sharing of the costs of WTI construction and operation. Part of the purpose of introducing private capital is to share the cost risk of the government’s monopoly on the construction and operation of infrastructure by exchanging the government’s appropriate concession of operating revenue^42^; however, excessive cost and risk sharing reduces the probability of private capital participation in the construction and operation of infrastructure^43^. In China, the return mechanisms of the public–private WTI include user payment, government payment, and feasibility gap subsidy, of which the cost risks of construction and operation under the first two return mechanisms are primarily borne unilaterally by the private capital and the government, respectively^44^, whereas the cost risks of construction and operation under the latter are borne by the government to fill the gap of user payment^39^. Accordingly, Hypothesis 2 is proposed:

The return mechanism of the feasibility gap subsidy has a greater impact on improving the UWTE than the mechanisms of user payment and government payment.

The way to choose private capital to cooperate with the government is related to the efficiency of the construction and operation of the WTI. Private capital selected through competitive procurement usually exhibits sufficient funds, scientific management experience, and innovative technology^45^. Cooperation between the government and this type of private partner helps obtain the optimal construction and operation plan at the lowest cost. The adoption of competitive procurement can improve efficiency while saving transaction costs, especially for infrastructures with large capital scale and long term and complicated operational systems, such as urban wastewater treatment^38^. In China, the competitive procurement mechanism of PPPs for WTI includes public bidding, competitive negotiation, invitational bidding, and competitive consultation, whereas the non-competitive procurement mechanism mainly refers to single-source procurement^46^. In accordance with this, Hypothesis 3 is proposed:

The competitive procurement mechanism has a greater impact on improving the UWTE than the single-source procurement mechanism.

The PPP is ultimately a contract between the principal and the agent that specifies how risks are shared and how benefits are distributed^40^. Construction and operation of WTI under the PPP model usually require long-term contracts. This means that contracts are often incomplete, and the allocation of remaining control rights has a significant impact on the incentives for private capital parties to participate. Existing research suggests that the greater the remaining control the private capital receives, the stronger their incentive to participate in the construction and operation of infrastructure, and the more they pursue innovation and efficiency^47^. The remaining control right is related to the manner in which the infrastructure is operated^48^. In China, PPPs for WTI operate through outsourcing (e.g. Operation and Maintenance [OM], Management Contract [MC], and Build-Transfer [BT]), franchising (e.g. Build-Operate-Transfer [BOT], Build-Own-Operate-Transfer [BOOT], Transfer-Operate-Transfer [TOT], and Rehabilitate-Operate-Transfer [ROT]), and privatisation (e.g. Build-Own-Operate [BOO] and Buy-Build-Operate [BBO]). Therefore, Hypothesis 4 is proposed:

Privatised operations have a greater impact on improving UWTE than outsourcing and franchising.

Promotion after demonstration has long been a feature of public policy formulation and implementation by the Chinese government, and this is also true for the construction and operation of wastewater treatment PPP projects. Selecting a portion of these projects for demonstration can facilitate pre-judgement of the issues encountered in the construction and operation of infrastructure and improve efficiency^49^. The demonstration of WTI is prioritised for various government policies and funding support and is subject to stringent monitoring by the government^50^. Therefore, to obtain priority support from the government, WTIs that have not entered the demonstration have greater motivation to perform higher quality wastewater treatment. In this case, Hypothesis 5 is proposed:

Wastewater treatment PPP projects that have not yet entered the demonstration have a UWTE higher than those that have been in the demonstration.

### Quantifying the UWTE using DEA

In order to measure the efficiency represented by the capacity to increase output at a given input, two methods have been proposed. One is the estimation method based on parameters. The common method is stochastic frontier analysis (SFA). The other is based on the nonparametric estimation method, and the DEA is the most widely used. Although SFA can consider the influence of random factors on output, it needs to determine the specific form of production frontier as the condition when measuring efficiency. This means that if the pre-set production function form is inconsistent with the reality, the efficiency of the measure is not accurate. In contrast, the advantage of DEA is that there is no need to presuppose a specific production function form. It is based on a number of input and output indicators, using the method of linear programming, with the data envelope frontier as the comparison base, the decision making unit (DMU) of the same type of relative evaluation to determine the efficiency. In addition, DEA can also give the improvement space of each DMU in terms of input and output, which is convenient to give optimisation suggestions. Thus, DEA is widely used to assess the efficiency of public services, the environment, and natural resources fields^51^. With different settings of comparative DMUs, DEA can be divided into the CCR model, which assumes that the comparative DMUs meet the condition of constant returns to scale, and the BCC model, which assumes that the comparative DMUs meet the condition of variable returns to scale, and Shephard distance function introduced to distinguish pure technical efficiency from scale efficiency and determine whether the DMU production is optimal. Most studies have concluded that the BCC model is more consistent with the reality of production^52^; therefore, it is widely accepted and adopted compared to the CCR model. In this study, DEA based on the BCC model was used to measure the UWTE. The length of the urban wastewater network and the daily treatment capacity of urban wastewater treatment plants are established as input indicators, and the total amount of urban wastewater treatment is established as the output indicator^53^. The efficiency for each DMU is measured by solving the following linear programming of the BCC model, shown in Eq. (1):

1 $$\begin{array}{l}\max \theta \\ s.t.\mathop {\sum }\limits_{i = 1}^{283} \lambda _i \cdot lwn_i \le lwn_{i_0}\\ \mathop {\sum }\limits_{i = 1}^{283} \lambda _i \cdot dtc_i \le dtc_{i_0}\\ \mathop {\sum }\limits_{i = 1}^{283} \lambda _i \cdot tawt_i \le \theta tawt_{i_0}\\ \lambda _i \ge 0\\ \mathop {\sum }\limits_{i = 1}^{283} \lambda _i = 1\end{array}$$

where subscript *θ* denotes the evaluated DMU. *lwn*_*i*_ and *dtc*_*i*_ represent the inputs, i.e. length of the wastewater network and the daily treatment capacity of urban wastewater treatment plants in prefecture-level city *i*, respectively, and the output is *tawt*_*i*_, the total amount of wastewater treatment of each prefecture-level city. is a *λ* vector of intensity variable, and *θ* represents the efficiency score based on the input-output calculation. This is the UWTE to be calculated in this study.

### Causal linking the PPPs to the UWTE using DEA-Tobit regression model

The DEA-Tobit regression model was used to empirically test the causal relationship between the PPPs and the UWTE. It is meaningful to use DEA to measure the UWTE, because the measured relative efficiency can be used to evaluate the capacity of urban wastewater treatment, and make it possible to compare the capacity of urban wastewater treatment between prefecture-level cities, and also creates conditions for finding the factors affecting the UWTE. As the range of UWTE measured by DEA is between 0 and 1, it does not obey the normal distribution and violates the classical assumption of ordinary least squares estimation. Therefore, in order to avoid the bias caused by OLS estimation, the restricted dependent variable model, also known as the Tobit regression model, is usually adopted in previous studies. The regression model which combines DEA and the Tobit regression model is also called the DEA-Tobit regression model. This study employs a DEA-Tobit regression model based on panel data, shown in Eq. (2).

2 $$uwte_{it} = \beta _0 + \beta _1 \cdot PPP_{it} + X^\prime \cdot \gamma + \varepsilon _{it}$$

where *uwte* denotes the efficiency of urban wastewater treatment. *PPP* denotes the degree of development of urban wastewater treatment PPP projects, which is measured in three calibres by determining the presence or absence of wastewater treatment PPP projects, the number of wastewater treatment PPP projects, and the investment amount of wastewater treatment PPP projects. *X*′ denotes other main control variables that potentially affect UWTE including population density, urbanisation rate, GDP per capita, industrialisation rate, openness, and green innovations. *i* and *t* represent prefecture-level city and year, respectively. *β*_0_ and *ε*_*it*_ denote the intercept term and the random disturbance term, respectively. *β*_1_ and *γ* are both parameters to be estimated, and *β*_1_ is significantly positive, indicating that the PPP model has a significant positive effect on the UWTE. Because the DEA-Tobit regression model with panel data does not have consistent and unbiased parameter estimates obtained under the fixed effects, the random effects estimation method is used in this study, referring to the parameter estimation recommendations presented by Liu et al.^54^

### Measurements of dependent, explanatory and control variables

The dependent variable in this study is the UWTE. As mentioned above, we use DEA based on the BCC model to measure the UWTE. The closer the value of UWTE is to 1, the higher the efficiency is; the closer it is to 0, the lower the efficiency is.

The degree of PPP development is the key explanatory variable of this study. It can be measured in various ways. The most common approach is determining the presence or absence of PPP projects, the number of PPP projects, and the investment amount of PPP projects^31,33^. To assess the impact of PPP on the UWTE in a comprehensive and reliable manner, this study uses all three metrics simultaneously.

The endogeneity of mutual causation must be addressed when investigating the causal relationship between PPPs and the UWTE. This is because prefecture-level cities that use PPP models to build and operate WTI may consider wastewater treatment to be important, for example, the promotion of local government officials is closely related to the quality of public services in their jurisdictions during their tenure. To obtain a higher promotion probability, these prefecture-level cities focus on the efficiency of urban public services, including wastewater treatment, and the higher UWTE determines their willingness to adopt PPPs. Therefore, this study uses instrumental variables to eliminate the endogeneity problem in the regression analysis.

Exogenous and correlation conditions are required for suitable instrumental variables. Waste treatment PPP development measured by determining the presence or absence and the number of waste treatment PPP projects is an instrumental variable for the degree of wastewater treatment PPP projects. This is because waste treatment and wastewater treatment are both urban environmental protection infrastructures. Furthermore, prefecture-level cities that consider wastewater treatment are highly likely to consider waste treatment, which are highly correlated. The PPP development for waste treatment does not directly affect the UWTE. Furthermore, the mean number of wastewater PPP projects in neighbouring prefecture-level cities in the prefecture-level city’s province was an instrumental variable for wastewater treatment PPP projects there. This is because, on the one hand, local government officials proactively follow the practices of other neighbouring prefecture-level cities in the province^55^. Assuming that other neighbouring prefecture-level cities in the province are inclined to promote wastewater treatment PPP projects, the prefecture-level city is highly likely to adopt a PPP model for the construction and operation of WTI. However, the mean number of wastewater treatment PPP projects in other neighbouring prefecture-level cities in the province will not directly affect UWTE in the prefecture-level city.

Control variables: based on IPAT theory^56^, population density, urbanisation rate, GDP per capita, industrialisation rate, openness, and green innovations were selected in this study to measure the influence of three dimensions of population, wealth, and technology on the UWTE. The population density is measured as the urban population divided by the urban area. The higher the population density, the greater the need for an urban wastewater treatment capacity. The urbanisation rate is calculated as the share of urban population in the total population of the prefecture-level city. The higher the urbanisation rate, the higher the population in urban areas and the higher the demand for urban wastewater treatment capacity. Meanwhile, the urban population produces relatively more wastewater.

GDP per capita is measured as GDP divided by population. The higher the GDP per capita, the higher the level of economic development of the prefecture-level city, and the more the government can regulate urban wastewater^35^, thus affecting the UWTE. The industrialisation rate is obtained by calculating the ratio of the output value of the secondary industry to GDP. The higher the industrialisation rate, the greater the demand for urban water resources, and more wastewater discharges are generated^2^, which affects the UWTE. Openness is measured by the proportion of imports and exports to GDP. The higher the openness, the more likely it is to attract companies with advanced environmental technologies^57^, reducing the amount of wastewater discharged from the prefecture-level city’s production sector. The ‘pollution heaven’ hypothesis may attract additional pollution discharge enterprises to the prefecture-level city^58^, affecting the prefecture-level city’s UWTE. Green innovations are measured using the number of green patents for wastewater treatment. Green patents for wastewater treatment are obtained from the Green List of International Patent Classification provided by the World Intellectual Property Organization (WIPO). If there are green patents for wastewater treatment, the reduction of wastewater discharge from enterprises is more likely, and thus the UWTE is improved^59^. This study considers the logarithm of the number of green patents for wastewater treatment to avoid the influence of data heteroscedasticity on the regression estimation results.

### Data

The research sample in this study comprised 1303 wastewater treatment PPP projects in 283 prefecture-level cities in China from 2014 to 2019, excluding Hong Kong, Macao, and Taiwan. To estimate the impact of PPPs on the UWTE, we needed data on the length of urban wastewater network, daily treatment capacity of urban wastewater treatment plants, total amount of urban wastewater treatment, wastewater treatment PPP projects, population density, urbanisation rate, GDP per capita, industrialisation rate, openness, and green innovations. Data on the length of the urban wastewater network, the daily treatment capacity of urban wastewater treatment plants, and the total amount of urban wastewater treatment were obtained from the China Urban Construction Statistical Yearbook 2014–2019^60^. The PPP data were obtained from the Ministry of Finance’s Public–Private Partnerships Center^61^ and were captured by python technology. Data on population density, urbanisation rate, GDP per capita, industrialisation rate, and openness were obtained from China City Statistical Yearbook 2015–2020^62^, and data on green patents were obtained from China National Intellectual Property Administration^63^. Supplementary Table 1 presents the descriptive statistics of the main variables, and Supplementary Fig. 1 reports the UWTE of 283 prefecture-level cities in China from 2014 to 2019.

## Supplementary information

Supplementary Information

## Data Availability

The data that support the findings of this study are publicly available at 10.6084/m9.figshare.21782582^64^.

## Code availability

STATA (16) is the major applications used to obtain the DEA-Tobit regression results. The codes for evaluating the impacts of public–private partnerships on the urban wastewater treatment efficiency in Chinese prefecture-level cities (2014–2019) are provided in the Supplementary Information.
