# Supplementary material for: Potential benefits of public–private partnerships to improve the efficiency of urban wastewater treatment
Source: NPJ Clean Water. 2023 Feb 20;6(1):13. doi: 10.1038/s41545-023-00232-2 (PMC9943046; doi:10.1038/s41545-023-00232-2)
Supplement: Supplementary file 1 — Supplementary Information [file 41545_2023_232_MOESM1_ESM.pdf]

Supplementary information for

Potential benefits of public–private partnerships to improve the efficiency of urban wastewater treatment

Shulei Cheng[1], Yu Yu[1], Fanxin Meng[2,*], Jiandong Chen[1], Yongtao Chen[3], Gengyuan Liu[2] and Wei Fan[4]

The supplementary information provides additional contents, including: (1) a representative literature review of the various tools used by governments worldwide to improve UWTE; (2) a representative literature review of the impacts of macro-institutions and policies on the UWTE; (3) representative evidence related to the effects of PPP on the running efficiency of other infrastructures; (4) a table that reported the descriptive statistics of variables; (5) a figure that reported the urban wastewater treatment efficiency (UWTE) in Chinese prefecture-level cities during 2014–2019; (6) codes for evaluating the impacts of public–private partnerships (PPPs) on the urban wastewater treatment efficiency (UWTE) in Chinese prefecture-level cities during 2014-2019.

[1.]School of Public Administration, Southwestern University of Finance and Economics, Chengdu 611130, China. [2.]State Key Joint Laboratory of Environment Simulation and Pollution Control, School of Environment, Beijing Normal University, Beijing 100875, China. [3.]School of Finance, Southwestern University of Finance and Economics, Chengdu 611130, China. [4.]School of Economics, Southwestern University of Finance and Economics, Chengdu 611130, China. [*]Corresponding author: email: fanxin.meng@bnu.edu.cn.

Contents

Supplementary Discussion 1 Representative literature review of the various tools used by governments worldwide to improve UWTE

Supplementary Discussion 2 Representative literature review of the impacts of macro-institutions and policies on the UWTE

Supplementary Discussion 3 Representative evidence related to the effects of PPP on the running efficiency of other infrastructures

Supplementary Table 1 Descriptive statistics of variables.

Supplementary Figure 1 Urban wastewater treatment efficiency (UWTE) in Chinese prefecture-level cities during 2014–2019.

Supplementary Note Codes for evaluating the impacts of public–private partnerships (PPPs) on the urban wastewater treatment efficiency (UWTE) in Chinese prefecture-level cities during 2014-2019.

Supplementary Discussion 1 Representative literature review of the various tools used by governments worldwide to improve UWTE

Governments worldwide use various tools, including penalties, subsidies, and administrative rules, to inhibit the discharge-based wastewater (including that from manufacturing industries and households). They strive to relieve the pressure of WTI on communities to purify urban wastewater and indirectly create conditions for improving UWTE. The government can either punish enterprises by monitoring whether they produce excessive wastewater [1]; they can give appropriate subsidies to enterprises using advanced wastewater purification technology and equipment [2]; the government can directly regulate the amount of wastewater discharged by enterprises by giving discharge permits [3]; or they can encourage households to reduce water use and wastewater through tiered water price subsidies [4].

Supplementary Discussion 2 Representative literature review of the impacts of macro-institutions and policies on the UWTE

Previous studies have focused on the impacts of macro-institutions and policies on the UWTE. A river chief system that optimises water management links the UWTE to the performance assessment of local government officials, causing WTIs to expand their wastewater transportation and treatment capacity [5]. Diversified financing mechanisms and professional management systems have become the accepted way for improving the UWTE, as they can solve the problems of technological innovation obstacles and insufficient employee incentives [6].

Considering the economies of scale existing in the WTI, it is easier for smaller cities to create community associations (achieving the minimum threshold for ensuring the UWTE) than larger cities, for example, sludge centralised management [7]. Owing to the gradual ageing of WTIs, preventive maintenance policy is also an important guarantee for improving the UWTE [8]. As the main source of wastewater discharged by production activities in urban areas, enterprises share and pay for wastewater treatment facilities constructed and operated by the government. This helps to address the excessive wastewater discharge caused by the agglomeration of various types of enterprises during the development of industrial parks. This phenomenon is becoming prevalent in certain regions with sufficient public budgets [9]. Eco-industrial parks, which aim to improve environmental, economic, and social performance through collaboration, have also been proven to be more helpful in overcoming excessive wastewater discharge caused by industrial clusters and improving the UWTE [10]. Therefore, the impacts of macro-institutions and policies on the UWTE are largely related to the construction and operation mode selected for the WTI.

Supplementary Discussion 3 Representative evidence related to the effects of PPP on the running efficiency of other infrastructures

In transportation, Fathi and Shrestha [11] found that as the PPP model has the advantage of fast-tracking project design and construction progress, highways constructed have a high average construction intensity and low cost and thus are more efficient. In energy services, Carbonara and Pellegrino [12] verified that as long as the

public sector and energy service enterprises are designed to equally share the benefits and adequate public procedures are established to support selecting the most appropriate energy performance contract under specific circumstances and project characteristics, the service efficiency using the PPP model can be improved. In public health, Sekhri et al. [13] found that the infrastructure built and operated by the PPP model can consider the accessibility, quality, and efficiency of healthcare as this model can not only improve the stability of capital and operating public budget, equity of access opportunities, and quality but can also accompany the establishment of an independent monitoring and evaluation body to ensure that performance is monitored using measurable and internationally recognised standards.

Supplementary Table 1 Descriptive statistics of variables

Variables	Measurements	Obs.	Mean	SD	Min	Max
UWTE	Urban wastewater treatment efficiency	1,698	0.63	0.17	0.07	1
PPP development						
PPP	Whether or not the prefecture-level city adopts the PPPs in wastewater treatment	1,698	0.39	0.49	0	1
PPPn	Number of wastewater treatment PPP projects	1,698	0.77	1.72	0	31
lnPPPinv	Logarithm form of the investment amount of wastewater treatment PPP projects	1,698	2.97	4.80	0	15.07
Prefecture-level city characteristics						
lngdp	Logarithm form of GDP divided by population	1,698	10.83	0.55	9.23	15.68
industrial	Ratio of the output value of the secondary industry to GDP	1,698	44.49	10.36	10.68	75.53
openness	Proportion of imports and exports to GDP	1,688	17.50	29.05	5.46e-05	262.90
popdensity	Urban population divided by the urban area	1,692	443.70	350.40	3.94	2,759
urban	Share of urban population in the total population of the prefecture-level city	1,683	56.31	14.17	21.83	100
lngreen	Logarithm form of the number of green patents in wastewater treatment	1,698	2.45	2.02	0	8.00

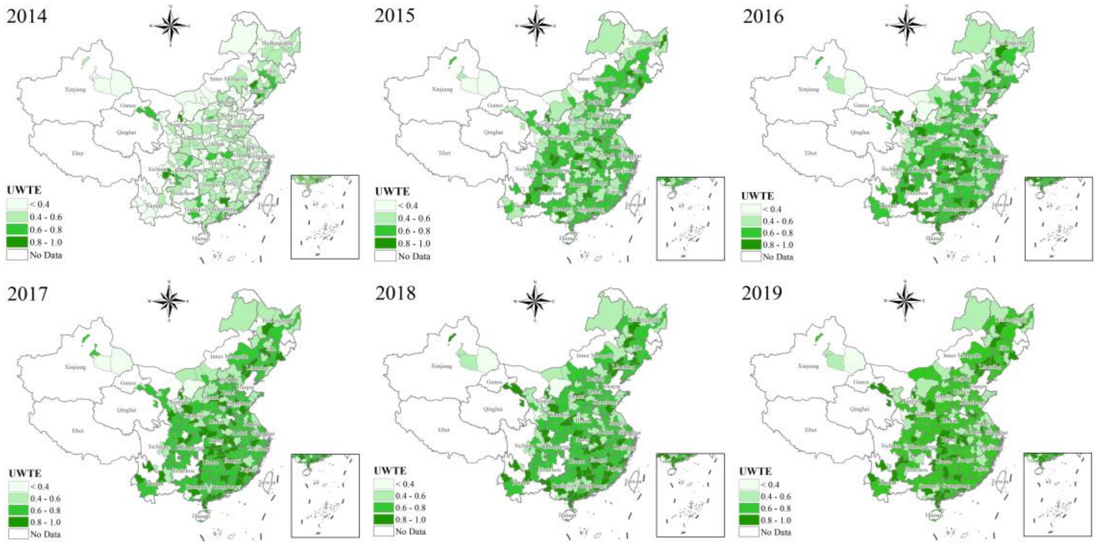

Supplementary Figure 1 Urban wastewater treatment efficiency (UWTE) in Chinese prefecture-level cities during 2014–2019. The number in the upper left corner of the figures represents the year. UWTE represents the urban wastewater treatment efficiency obtained by data envelopment analysis, and the colour from light to dark represents the efficiency from low to high. The critical value takes the colour which belongs to the higher interval. The division of research area is based on prefecture-level cities. The uncoloured prefecture-level cities lack data on efficiency calculations.

Supplementary Note Codes for evaluating the impacts of public–private partnerships (PPPs) on the urban wastewater treatment efficiency (UWTE) in Chinese prefecture-level cities during 2014-2019

```
*Potential benefits of public-private partnerships for improving urban wastewater treatment efficiency
using data dea-tobit.dta,clear
* Table 1: Estimation results of benchmark regression model
xtset idcode year
gen ppp = 0 if !missing(ppp_dummy)
replace ppp = 1 if ppp_dummy ==1 & !missing(ppp_dummy)
xttobit crste ppp ,ul(1) tobit
margins, dydx(*)
estimates store a1
xttobit crste ppp popdensity urban lngdp industrial openness lngreen ,  ul(1) tobit
margins, dydx(*)
estimates store a2
xttobit crste pppn,ul(1) tobit
margins, dydx(*)
estimates store a3
xttobit crste pppn popdensity urban lngdp industrial openness lngreen,ul(1) tobit
margins, dydx(*)
estimates store a4
esttab a1 a2 a3 a4 using table1.rtf ,b(%6.3f) se(%6.3f) star(* 0.1 ** 0.05 *** 0.01) replace
using data iv.dta,clear
*Table 2: Estimation results of instrument variable regression model
xtset idcode year
gen ppp = 0 if !missing(ppp_dummy)
replace ppp = 1 if ppp_dummy ==1 & !missing(ppp_dummy)
```

```
generate y1_ll = crste
replace y1_ll = . if crste<=0
generate y1_ul = crste
replace y1_ul = . if crste>=1 & crste<.
eintreg y1_ll y1_ul popdensity urban lngdp industrial openness lngreen,
endogenous(ppp = wasteppp lngdp industrial openness popdensity urban
lngreen,probit)
estimates store b1
reg ppp wasteppp popdensity urban lngdp industrial openness lngreen
estimates store b2
ivtobit crste popdensity urban lngdp industrial openness lngreen (pppn=wastepppn),
ul(1) first
estimates store b3
reg pppn wastepppn popdensity urban lngdp industrial openness lngreen
estimates store b4
bysort year province :  egen  total_number=total(pppn)
bysort year province :  egen  number1=count(pppn)
gen  deps=total_number-pppn
gen  meanpppn=deps/(number1-1)
ivtobit crste popdensity urban lngdp industrial openness lngreen (pppn=meanpppn),
ul(1) first
estimates store b5
reg pppn meanpppn popdensity urban lngdp industrial openness lngreen
estimates store b6
esttab b1 b2 b3 b4 b5 b6 using table2.rtf ,b(%6.3f) se(%6.3f) star(* 0.1 ** 0.05 ***
0.01) replace
*Table 3: Robustness check results
xttobit crste lnpppinv ,ul(1) tobit
margins, dydx(*)
estimates store c1
```

```
xttobit crste lnpppinv popdensity urban lngdp industrial openness lngreen,ul(1) tobit
margins, dydx(*)
estimates store c2
bysort year province :  egen  total_number1=total(pppn)
bysort year province :  egen  number2=count(pppn)
gen  meanpppn1=total_number1/number2
bysort year province :  egen  total_lninv11=total(lnpppinv)
bysort year province :  egen  geshu1=count(lnpppinv)
gen  meanpppinv=total_lninv11/geshu1
ivtobit crste popdensity urban lngdp industrial openness lngreen (pppn=meanpppn1),
ul(1) first
estimates store c3
reg pppn meanpppn1 popdensity urban lngdp industrial openness lngreen
estimates store c4
ivtobit crste popdensity urban lngdp industrial openness lngreen
(lnpppinv=meanpppinv), ul(1) first
estimates store c5
reg lnpppinv meanpppinv popdensity urban lngdp industrial openness lngreen
estimates store c6
ivtobit crste popdensity urban lngdp industrial openness lngreen
(lnpppinv=wastepppinv), ul(1) first
estimates store c7
reg lnpppinv wastepppinv popdensity urban lngdp industrial openness lngreen
estimates store c8
esttab c1 c2 c3 c4 c5 c6 c7 c8 using table3.rtf ,b(%6.3f) se(%6.3f) star(* 0.1 ** 0.05
*** 0.01) replace
using data tobit.dta,clear
*Table 4: Effect of return mechanism
xtset idcode year
gen ppp = 0 if !missing(ppp_dummy)
```

```
replace ppp = 1 if ppp_dummy ==1 & !missing(ppp_dummy)
xttobit crste feasibilitygapsubsidy popdensity urban lngdp industrial openness lngreen,ul(1) tobit
margins, dydx(*)
estimates store d1
xttobit crste userpayment popdensity urban lngdp industrial openness lngreen,ul(1) tobit
margins, dydx(*)
estimates store d2
xttobit crste governmentpayment popdensity urban lngdp industrial openness lngreen,ul(1) tobit
margins, dydx(*)
estimates store d3
esttab d1 d2 d3 using table4.rtf ,b(%6.3f) se(%6.3f) star(* 0.1 ** 0.05 *** 0.01) replace
*Table 5: Effect of procurement mechanism
xttobit crste singlesourceprocurement popdensity urban lngdp industrial openness lngreen,ul(1) tobit
margins, dydx(*)
estimates store e1
xttobit crste competitiveprocurement popdensity urban lngdp industrial openness lngreen,ul(1) tobit
margins, dydx(*)
estimates store e2
esttab e1 e2 using table5.rtf ,b(%6.3f) se(%6.3f) star(* 0.1 ** 0.05 *** 0.01) replace
*Table 6: Effect of operation mechanism
xttobit crste outsourcing popdensity urban lngdp industrial openness lngreen,ul(1) tobit
margins, dydx(*)
estimates store f1
```

```
xttobit crste franchising popdensity urban lngdp industrial openness lngreen,ul(1) tobit
margins, dydx(*)
estimates store f2
xttobit crste privatizedoperation popdensity urban lngdp industrial openness lngreen,ul(1) tobit
margins, dydx(*)
estimates store f3
esttab f1 f2 f3 using table6.rtf ,b(%6.3f) se(%6.3f) star(* 0.1 ** 0.05 *** 0.01) replace
*Table 7: Effect of demonstration
gen nondemonstration=pppn-demonstration
xttobit crste nondemonstration popdensity urban lngdp industrial openness lngreen,ul(1) tobit
margins, dydx(*)
estimates store g1
xttobit crste demonstration popdensity urban lngdp industrial openness lngreen,ul(1) tobit
margins, dydx(*)
estimates store g2
esttab g1 g2 using table7.rtf ,b(%6.3f) se(%6.3f) star(* 0.1 ** 0.05 *** 0.01) replace
using heterogeneous.dta,clear
*Table 8: Heterogeneous analysis
*area
xtset idcode year
gen ppp = 0 if !missing(ppp_dummy)
replace ppp = 1 if ppp_dummy ==1 & !missing(ppp_dummy)
xttobit crste pppn popdensity urban lngdp industrial openness lngreen if area==2 ,ul(1) tobit
margins, dydx(*)
```

```
estimates store h1
xttobit crste pppn popdensity urban lngdp industrial openness lngreen if area==1 ,ul(1) tobit
margins, dydx(*)
estimates store h2
xttobit crste pppn popdensity urban lngdp industrial openness lngreen if area==3 ,ul(1) tobit
margins, dydx(*)
estimates store h3
*market
xttobit crste pppn popdensity urban lngdp industrial openness lngreen if market==1 ,ul(1) tobit
margins, dydx(*)
estimates store h4
xttobit crste pppn popdensity urban lngdp industrial openness lngreen if market==2 ,ul(1) tobit
margins, dydx(*)
estimates store h5
*rain
xttobit crste pppn popdensity urban lngdp industrial openness lngreen if rain==1 ,ul(1) tobit
margins, dydx(*)
estimates store h6
xttobit crste pppn popdensity urban lngdp industrial openness lngreen if rain==2 ,ul(1) tobit
margins, dydx(*)
estimates store h7
esttab h1 h2 h3 h4 h5 h6 h7 using table8.rtf ,b(%6.3f) se(%6.3f) star(* 0.1 ** 0.05 *** 0.01) replace
* Table S1: Descriptions of variables and summary statistics
```

```
global var "ppp pppn lnpppivn popdensity urban lngdp industrial openness lngreen"
outreg2 using S1.rtf, replace sum(log) keep(**$var**) title(Descriptive statistics)
```

Supplementary references

1 Greenstone, M. & Hanna, R. Environmental regulations, air and water pollution, and infant mortality in India. *Am. Econ. Rev.* 1**04(10)**, 3038–3072 (2014).

2 Sauer, P. *et al.* Improving quality of surface waters with coalition projects and environmental subsidy negotiation. *Pol. J. Environ. Stud.* **24(3),** 1299–1307 (2015).

3 Hernández-Chover, V., Bellver-Domingo, A. & Hernández-Sancho, F. Efficiency of wastewater treatment facilities: the influence of scale economies. *J. Environ. Manag.* **228,** 77–84 (2018).

4 Mombeni, H. A., Rezaei, S., Nadarajah, S. & Emami, M. Reducing water consumption after targeted subsidy plan in Iran. *Water Resour.* **42(3),** 389–396 (2015).

5 Zhang, Z. X. *et al.* Investigating the spatiotemporal dynamic evolution and driving factors of wastewater treatment efficiency in the context of China's River Chief system. *Ecol. Indic.* **129,** 107991 (2021).

6 Vélez-Ramírez, A., Rivera-Castañeda, P. & Muñoz-Pizza, D. M. Institutional capacity determinants in a global south city: the case of a wastewater utility in Zacatecas, Mexico. *Util. Policy* **79,** 101453 (2022).

7 Hernández-Chover, V., Bellver-Domingo, A. & Hernández-Sancho, F. Efficiency of wastewater treatment facilities: the influence of scale economies. *J. Environ. Manag.* **228,** 77–84 (2018).

8 Hernández-Chover, V., Castellet-Viciano, L. & Hernández-Sancho, F. Preventive maintenance versus cost of repairs in asset management: an efficiency analysis in wastewater treatment plants. *Process Saf. Environ.* **141,** 215–221 (2020).

9 Yuan, Z. W., Zhang, L. & Bi, J. Which is more cost-effective? A comparison of two wastewater treatment models in China-Singapore Suzhou Industrial Park, China. *J. Clean. Prod.* **18(13),** 1270–1275 (2010).

10 Hou, S. *et al.* Spatial analysis connects excess water pollution discharge, industrial production, and consumption at the sectoral level. *npj Clean Water* **5**, 4 (2022).

11 Fathi, M. & Shrestha, P. P. Public-private partnership project performance aanalysis compared to design-build in highway projects. *J. Constr. Eng. M.* **148(11),** 04022118 (2022).

12 Carbonara, N. & Pellegrino, R. Public-private partnerships for energy efficiency projects: a win-win model to choose the energy performance contracting structure. *J. Clean. Prod.* **170,** 1064–1075 (2018).

13 Sekhri, N., Feachem, R. & Ni, A. Public-private integrated partnerships demonstrate: the potential to improve health care access, quality, and efficiency. *Health Affair.* **30(8),** 1498–1507 (2011).
